# A technical guide to TRITEX, a computational pipeline for chromosome-scale sequence assembly of plant genomes

**DOI:** 10.1186/s13007-022-00964-1

**Published:** 2022-12-02

**Authors:** Marina Püpke Marone, Harmeet Chawla Singh, Curtis J. Pozniak, Martin Mascher

**Affiliations:** 1 Leibniz-Institute of Plant Genetics and Crop Plant Research (IPK), Gatersleben, Seeland, Germany; 2 Department of Genetics, Evolution, Microbiology and Immunology, University of Campinas, Campinas, Brazil; 3 Crop Development Centre and Department of Plant Sciences, University of Saskatchewan, Saskatoon, SK S7N 5A8 Canada; 4 Department of Plant Science, University of Manitoba, Winnipeg, MB R3T 2N2 Canada; 5 German Centre for Integrative Biodiversity Research (iDiv) Halle-Jena-Leipzig, Leipzig, Germany

**Keywords:** Genome sequence assembly, Chromosome conformation capture sequencing, Long-read sequence assembly, Genetic map, Pangenome

## Abstract

**Background:**

As complete and accurate genome sequences are becoming easier to obtain, more researchers wish to get one or more of them to support their research endeavors. Reliable and well-documented sequence assembly workflows find use in reference or pangenome projects.

**Results:**

We describe modifications to the TRITEX genome assembly workflow motivated by the rise of fast and easy long-read contig assembly of inbred plant genomes and the routine deployment of the toolchains in pangenome projects. New features include the use as surrogates of or complements to dense genetic maps and the introduction of user-editable tables to make the curation of contig placements easier and more intuitive.

**Conclusion:**

Even maximally contiguous sequence assemblies of the telomere-to-telomere sort, and to a yet greater extent, the fragmented kind require validation, correction, and comparison to reference standards. As pangenomics is burgeoning, these tasks are bound to become more widespread and TRITEX is one tool to get them done. This technical guide is supported by a step-by-step computational tutorial accessible under https://tritexassembly.bitbucket.io/. The TRITEX source code is hosted under this URL: https://bitbucket.org/tritexassembly.

**Supplementary Information:**

The online version contains supplementary material available at 10.1186/s13007-022-00964-1.

## Background

Sequences of plant genomes have been considered hard to assemble because of large genome sizes, high ploidy levels, and high repeat contents. In the past 3 years, genome sequencing and assembly methods have progressed so far as to make the assembly of inbred diploid genomes a routine task that can be scaled to the level of the pangenomes—chromosome-scale sequence assemblies of tens to hundreds of individuals of a species. Moderate hardware resources (50 CPU cores, 500 GB RAM) suffice to complete within hours the sequence assembly of a single homozygous multi-gigabase plant genome from accurate long-reads obtained using the PacBio HiFi method and state-of-the-art algorithms [1–3]. Oxford Nanopore (ONT) long reads have also been used as input for pseudomolecule construction and comparative evaluation [2]. In the future, highly accurate reads on the ONT platform (Q20+) may rival HiFi reads as the primary input for TRITEX. Universal solutions have yet to be devised to separate haplotype phases of heterozygous diploid or autopolyploid genomes. Promising results have been obtained in potato, an autotetraploid crop with a rather small haploid genome size of 1 Gb [4–6]. Strategies and tools to obtain haplotype-resolved assemblies have been reviewed in detail elsewhere [7, 8]. The assembly even of long and accurate sequence reads rarely results in sequence contigs spanning entire chromosomes from telomere to telomere [9]. Complementary linkage information is needed to arrange contigs into chromosome-scale scaffolds. The three most commonly used types of linkage information are genetic maps [10], optical maps [11], and chromosome conformation capture sequencing (Hi-C) data [12, 13]. Once a genome sequence assembly of a single representative individual of a species, the “reference genome”, has been obtained, reference-guide approaches premised on the high degree of collinearity of genomes of a single species, are applicable [14, 15].

Indeed, rather than relenting, sequence and assembly efforts tend to grow more intense in research communities with a seed capital of genomic sequences and means how to construct them of proven reliability. Pangenomes, collections of genome sequences of multiple individuals of a species, have been often and somewhat self-servingly heralded by genome scientists as an indispensable tool to understand genetic variation in many biological systems—a promise that seems so far to have been made good on. Pangenomes of cereal crops have revealed hitherto unknown variants linked with agronomic species and “super” or genus-wide pangenomes of crops and their wild relatives [16, 17]. We note that the word “pangenome” can refer to either a biological entity (the collection of DNA present in all individuals of a species) or a data structure (information comprising the genome sequences) representing (part of) the biological entity. The latter sense may more precisely capture by “pangenome infrastructure”. In the following, we use “pangenome” to refer to the data structure.

Conceptually, pangenome sequence assembly is not different from the reference kind, but only more of the same stuff. We argued in Mascher et al. [2] that the most cost-efficient approach for constructing tens of chromosome-scale cereal genome sequences is the same as used in constructing one barley reference genome: scaffolding contigs assembled from accurate long-reads with Hi-C linkage data. This approach has been pursued to construct the genus-wide pangenome of potato wild relatives [18].

One tool implementing the HiFi + HiC procedure is TRITEX. The first version of TRITEX [19], geared towards short-read data, was used to assemble one or more genome sequences of barley [2, 20], wheat [17], rye [21], oat [22], and eggplant [23], as well as the wheat wild relatives *Aegilops tauschii *[24], *Ae. sharonensis* [25], *Ae. longissima* and *Ae. speltoides* [26]. Here, we report on the things we have changed in and added to TRITEX since its first release in order to deal with much improved input sequence assemblies from accurate long reads and guide maps informed by now ubiquitous, near-perfect reference genomes. To illustrate the workings of TRITEX, we ran it on a publicly available maize dataset, on which we evaluated the impact of Hi-C link and guide map density.

## Results

### Overview of the TRITEX workflow

**Fig. 1:**
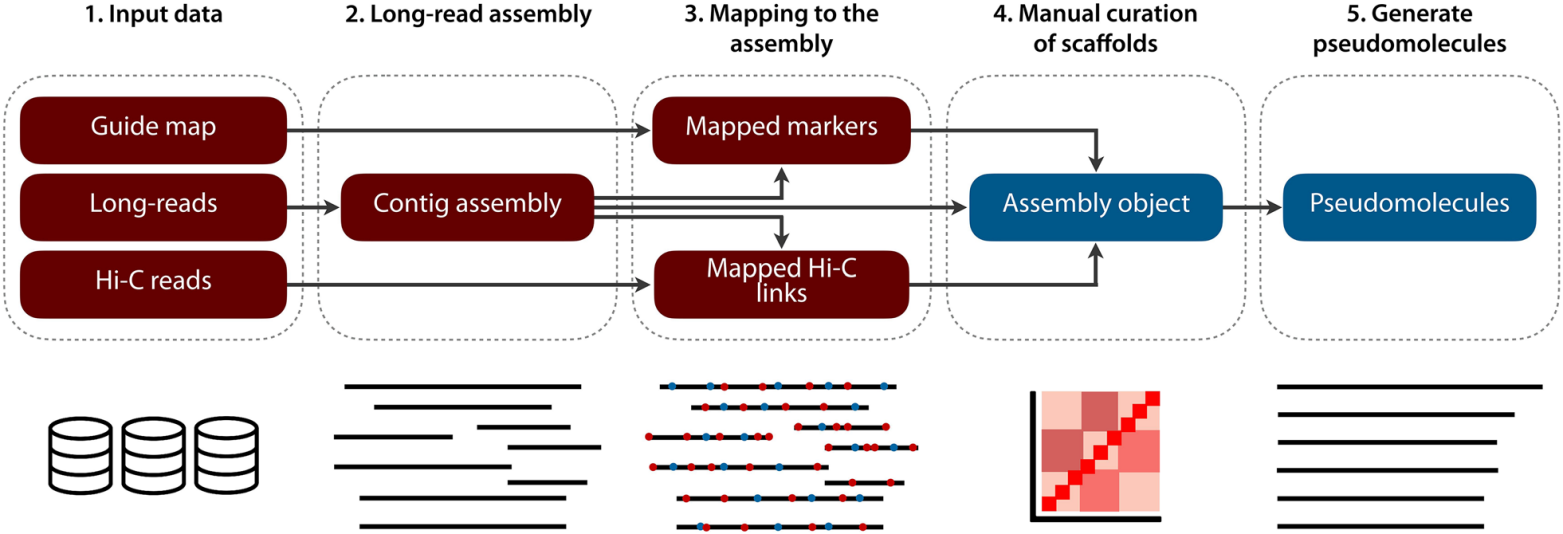
Graphical overview of the TRITEX pipeline. Steps in red boxes are done with Unix shell scripts; those in blue boxes on the R prompt

TRITEX is a computational pipeline for plant genome sequence assembly pipeline. It uses an input sequence assembly, Hi-C data, and a guide map to construct pseudomolecules, i.e. in silico representatives of entire chromosomes (Fig. 1). Contigs and Hi-C are well-known datatypes in contemporary sequence analysis. The concept of the guide map is more bespoke. It is a set of “markers”—sequence tags of variable length that are arranged in a linear order along the chromosomes. In contrast to other workflows such as 3D-DNA [27], TRITEX does not use Hi-C data to partition sequences into chromosomes. Instead, guide maps lift an existing solution to the problem of assigning sequences to chromosomes to a new assembly. In using guide maps, we borrow conceptually from reference-guided assembly methods such as RaGOO and RagTag [14, 15]. TRITEX also uses the guide map to constrain the Hi-C map while allowing minor perturbations relative to the reference to accommodate structural variation. In the original TRITEX approach, the guide map was a dense genome-wide genetic map. We have since extended TRITEX to work with guide maps derived from a reference genome.

TRITEX operates on sequence assemblies, the constituent parts of which we refer to as “scaffolds”. The initial short-read TRITEX included a complex, multi-step toolchain to assemble primary sequence contigs from short reads and scaffold them with mate-pairs and linked reads. This procedure has since been rendered obsolete by the rise of accurate long-read assembly, which is easier, faster and more powerful than short-read assembly [2]. For instance, the assembly of HiFi reads is a single-step process that can be accomplished by doing no more than running a single command. Other types of accurate long-reads, such as ONT Q20+, may underpin contig assembly with similar ease. The resultant sequences are in most cases contigs, i.e. contiguous sequences without gaps, and often span tens of megabases. For these reasons, primary sequence assembly is not a focus of TRITEX anymore, which has in the era of long-read assemblies become a tool for chromosome-scale scaffolding and is agnostic about which programs are used to assemble contigs as long as the latter are contiguous and complete enough to scaffold them with Hi-C.

The TRITEX workflow can be broadly divided into two stages. In the first, the user runs shell scripts combining standard bioinformatics tools such as the read-mapper minimap2 [28] and the alignment record processors SAMtools [29] and BEDTools [30] into a pipeline for processing Hi-C reads and aligning guide map markers. The second phase is done interactively at the prompt of the R statistical environment [31]. The outputs of phase 1 are read into main memory and a TRITEX assembly object with tables listing Hi-C links and guide map alignment records is initiated. The core algorithm for Hi-C map construction searches for a minimum spanning tree in the graph induced by Hi-C contact matrix and further refines it to include as many scaffolds as possible and to orient them relative to the chromosomal orientation of the guide map. The algorithm has been described in detail by Beier et al. [32] and has been unaffected by the changes brought about by much improved input assemblies and denser guide maps.

Once the assembly R object has been set up, Hi-C-based pseudomolecule construction can be proceeded with immediately. However, it is advisable before doing so to scrutinize the assembly for any obviously chimeric scaffolds, which join together sequences that are far apart in the actual genome. TRITEX provides static and interactive visualizations to help users display chimeric scaffolds produced in the input long-read assembly (with HiFi reads) and spot misplaced inverted scaffolds in the contact matrices. Static “diagnostic plots” show Hi-C coverage along the lengths of scaffolds as well as the collinearity of scaffolds to the guide map. Breakpoints in chimeras, i.e. the boundaries between falsely joined sequences, often co-colocalize with drops in physical Hi-C coverage (the number of Hi-C links spanning a genomic window). Diagnostic plots also show guide map alignments. If sequences from different chromosomes are joined, the chimeric scaffolds bear guide map markers from more than one chromosome (Additional file 1: Fig. S1). Intra-chromosomal chimeras also have markers from spatially separated regions, but this pattern is shared with true structural variations.

To come up with a list of putatively chimeric scaffolds, we use a simple heuristic: we look for scaffolds where Hi-C coverage falls by an adjustable threshold. The default setting is that coverage is at least eight-fold below the scaffold-wide average in internal regions (less than 100 kb away from the scaffold ends). Alternatively, users can generate diagnostic plots for all contigs. Highly contiguous long-read assemblies may consist of as little as tens of contigs, which can be inspected in a reasonable amount of time. Assuming a skilled user needs 2 s to spot a chimera, going through hundred contigs in a PDF viewer takes about 3 min. Troughs in Hi-C coverage tend to be shallower the closer two mis-joined sequences are to each other. Wrongly adjoining sequences that are not that far apart in reality (separated by less than 2% of the length of the chromosome) may result in only small disturbances of Hi-C coverage that may be spotted only by comparison to a high-density guide map or as off-diagonal signals in the contact matrix (see below).

To break chimeric scaffolds, users have to manually specify the coordinates of breakpoints to the TRITEX functions that update the assembly object so that Hi-C links and guide map alignment refer to corrected coordinates in the newly broken scaffolds. Often not all chimeras can be spotted from the get-go in diagnostic plots. Rather, cycles for chimera breaking, Hi-C map construction, and visual inspection of contact matrices may need to be repeated several times. Once a Hi-C map has been completed, the contact matrix, i.e. a heatmap showing the number of Hi-C links between genomic windows of fixed size (1 Mb by default), misplaced or misoriented scaffolds become manifest as off-diagonal signals (Additional file 1: Fig. S2). To help the inspection of contact matrices, we developed the Hi-C map inspector, an R Shiny App that is accessible through a web browser (Additional file 1: Fig. S2) after deployment on an R Shiny server. Genomic regions containing putatively chimeric scaffolds can be clicked on to get their names, create diagnostic plots for them and pinpoint the sites of culpable misjoins.

**Fig. 2:**
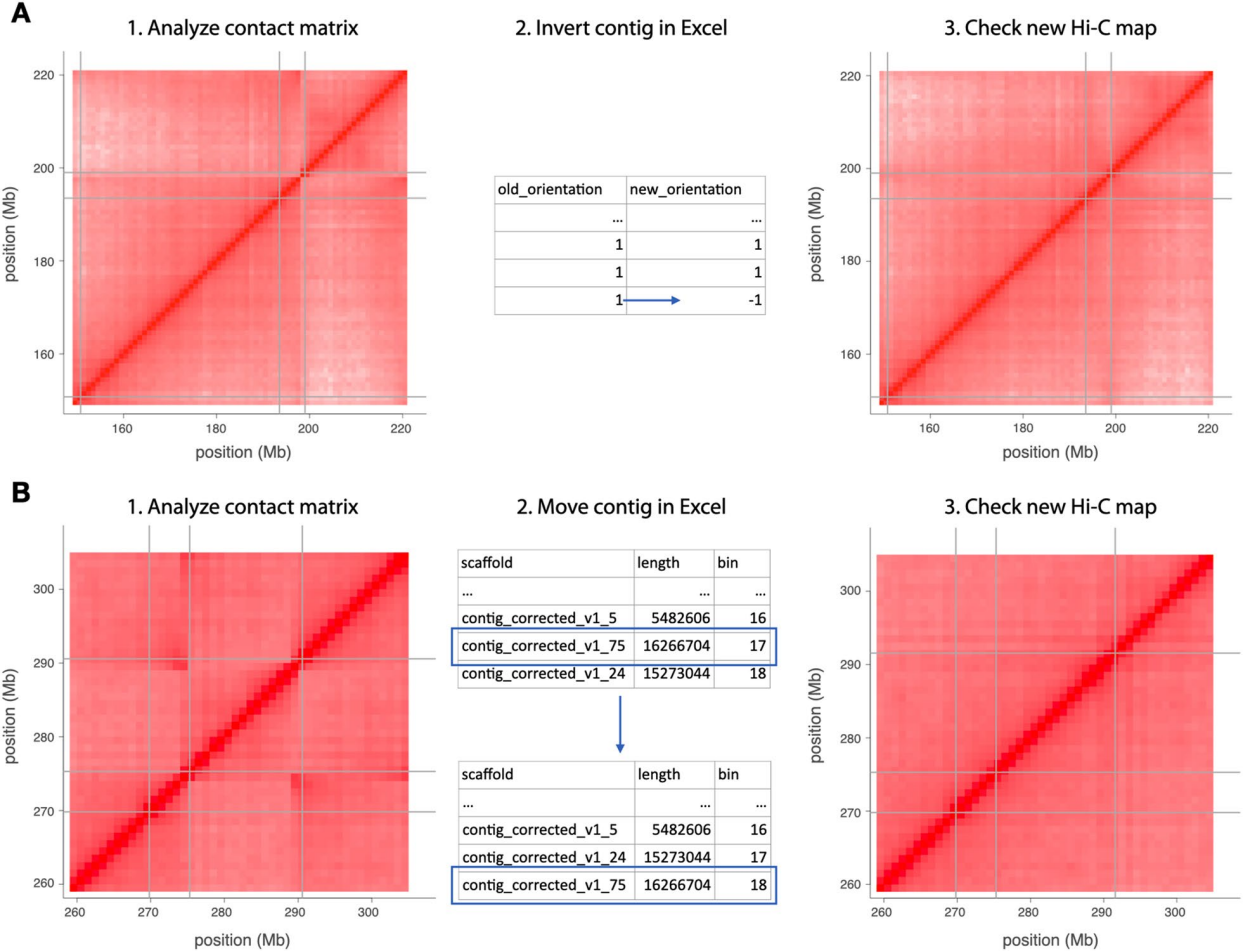
Manual curation in the TRITEX’s correct-map-inspect cycle. **A** The Hi-C contacts show a pattern indicative of an inversion in the terminal contig. The orientation is swapped in the Excel table and a new Hi-C matrix is computed with the updated configuration. The revised Hi-C matrix has fewer off-diagonal signals. **B** Hi-C contacts show a pattern indicative of a misplaced contig. The order of the final two rows is reversed in the Excel table and the Hi-C matrix is computed with the new configuration. The revised Hi-C matrix has fewer off-diagonal signals

Even if errors are absent from scaffolds, the Hi-C map constructed from them may have some. Oft-seen mistakes are wrongly oriented (“flipped”) scaffolds or groups of scaffolds that were flipped (Fig. 2A) or inserted in the wrong places (Fig. 2B). To correct this, we devised a method to manually edit Hi-C maps as ordered lists of contigs with their orientations in a spreadsheet application. One such application is Microsoft Excel, whose overzealous autocorrection unadapted to genomic sequence identifiers poses certain risks [33], which we believe in the present case to be offset by the ease of editing in a graphical user interface most people are accustomed to. One measure of risk mitigation is that after editing, tables are read into R, and even inconspicuously misformatted rows will throw errors. Formally valid, but biologically wrong edits will become evident in the comparison of contact matrices before and after editing. Users will know that their edits must have begot the oddities and can repeat the step.

After one or more correct-map-inspect cycles to set aright chimeras or Hi-C map glitches, the map is written out and “compiled”, meaning an AGP (“a golden path”) tabular file recording contig positions is written to disk and used together with the sequence file to piece together a FASTA file of the pseudomolecules. The scripts doing this are no different in principle from those in short-read TRITEX but are now much simpler, as the complex, multi-step scaffolding was retired. One noteworthy change is that sequences of unplaced scaffolds are now written to a multi-FASTA file with one sequence record for each of them instead of a single concatenated sequence (“chrUn”) because the latter format is refused by the archive of the International Nucleotide Sequence Database Collaboration. If needed, one could evaluate the final assembly to look for misassemblies by aligning the pseudomolecules to related species.

After this walkthrough, we illustrate some aspects of our pipeline with an example dataset for the maize inbred lines B73, which we also use to assess the impact of less dense linkage information on Hi-C map construction.

### TRITEX as run on a maize dataset

We downloaded HiFi and Hi-C reads of maize B73 from public archives. Assembly of the Hi-C reads with hifiasm yielded 928 contigs with an N50 of 37.8 Mb (Table 1) and a total length of 2.17 Gb, reasonably close to the flow-cytometric genome size estimate reported by Arumuganathan and Earle [34]. Out of a total of 859 million Hi-C read pairs, 89 million mapped with some degree of uniqueness (Q10 or better) to the assembly. The hifiasm assembly had three easily found chimeras (Additional file 1: Fig. S1) and no other surfaced in later steps.

**Table 1:** HiFi assembly statistics for maize B73

Number of contigs	928
Total length	2,176,313,099 bp
N50	37,817,658 bp
N90	7,282,328 bp
Mean contig length	2,345,164 bp
Maximum contig size	153,870,576 bp
Minimum contig size	14,160 bp

We constructed two guide maps. The first, which we refer to as the “reference” map, was built from the maize RefGen_v5 reference genome sequence assembly [35] and mimics the density and resolution afforded to pangenome projects that add more sequence assemblies of diverse germplasm to an existing reference genome. We used the “mask_assembly.zsh” script included in TRITEX to extract from the reference pseudomolecules 538,812 single-copy sequences 100 bp or longer as described by Jayakodi et al. [16]. The second guide map, referred to as “the genetic map one”, was a linkage map [36] of the Intermated B73xMo17 (IBM) population with 3686 SNP markers, which are defined as 100-bp tags positioned on the maize AGPv1 [37]. If a species does not have a genome assembly yet, genetic maps are often the best genomic coordinate system to position sequences in a genomic infrastructure under construction and the use of the IBM map to re-assemble the maize genome illustrates the performance of TRITEX for de novo genome assembly.

**Fig. 3:**
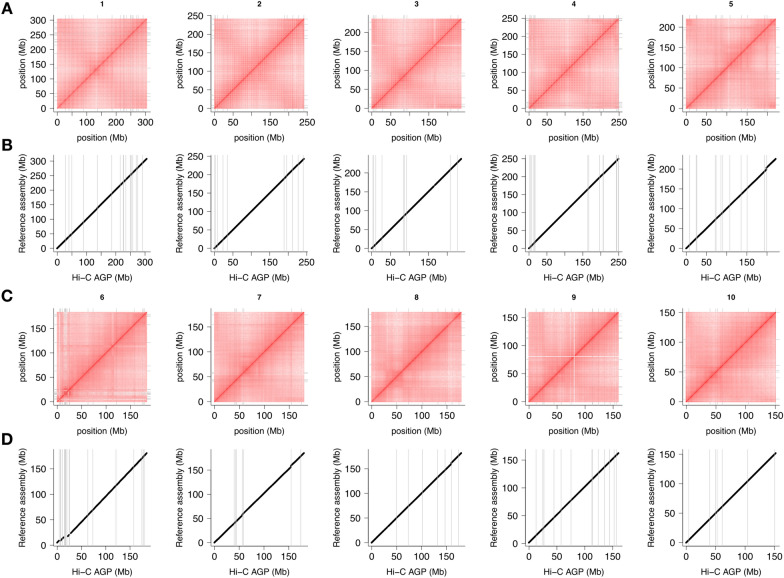
Hi-C contact matrices of reference-guided pseudomolecules (**A** and **C**) and collinearity of guide map markers to the maize B73 RefGen_v5 reference genome sequence (**B** and **D**). Grey lines mark contig boundaries

**Fig. 4:**
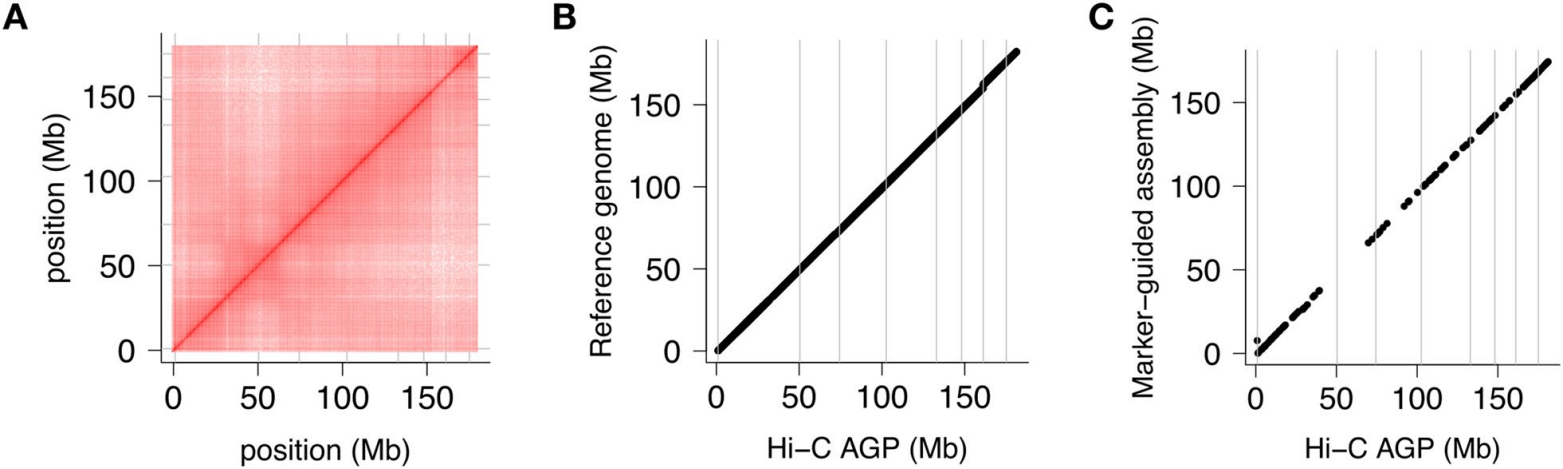
Marker-guided Hi-C map construction. Contact matrix (**A**), collinearity with the maize B73 RefGen_v5 reference genome sequence (**B**), and collinearity with the IBM guide map (**C**) for chromosome 8. Analogous plots for other chromosomes are shown in Additional file 1: Fig. S4

The reference and genetic guide maps yielded nearly the same Hi-C maps and assigned the vast majority of the assembled sequence to chromosomal locations. Hi-C contact matrices and alignment to the B73 RefGen_v5 reference (Figs. 3 and 4) confirmed the integrity of the pseudomolecules. Manual curation of the reference-guided assembly resolved a few inversions at chromosome termini. Uncommon signals decorated the interval 10–40 Mb on chromosome 6, which was composed of many small contigs. That region contains a highly repetitive ribosomal DNA locus (Additional file 1: Fig. S3), explaining the shortcomings of the contig assembly and the oddities in the Hi-C matrix. A region on chromosome 9 had very few Hi-C links, presumably because of its high repeat content and the attendant difficulties in mapping short Hi-C reads (Additional file 1: Fig. S4). As the region sat in the middle of a larger contig and Hi-C signals in its flanking regions are not out of the ordinary, a misassembly can be ruled out.

With the initial Hi-C matrices that were guided by a genetic map, manual curation was more involved and required more correct-map-inspect cycles because of the relative paucity of markers (Fig. 4C, chromosome 4 in Additional file 1: Fig. S4). Some smaller contigs (< 5 Mb) were assigned to chromosomes 3 and 4 by genetic markers whereas the reference-based correctly placed them on chromosome 6. We moved them to the unplaced scaffolds as without knowledge gleaned from the reference genome, we could not have placed them correctly. Even so, the final product was about as good as the reference-based Hi-C map (Table 2).

**Table 2:** Pseudomolecule statistics

	Reference	Genetic map
No. of contigs (chromosomes + unanchored)	10 + 808	10 + 810
Length pseudomolecules (bp)	2,121,154,572	2,114,179,800
Length of unanchored contigs (bp)	55,170,127	62,144,699
N50 (Mb)	222	222

### Can we scrimp on Hi-C?

**Fig. 5:**
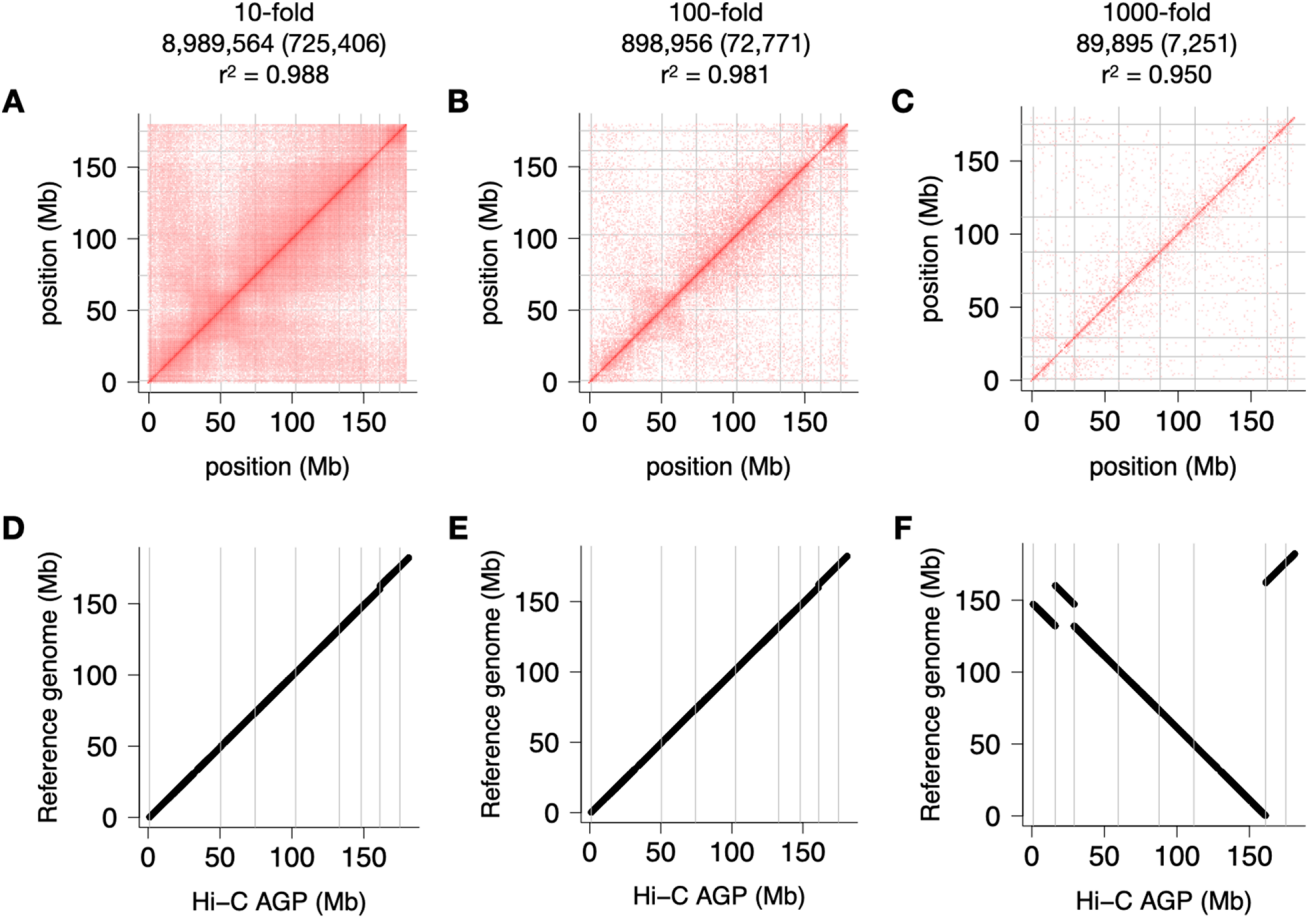
Impact of Hi-C data density on assembly quality. Hi-C maps were constructed from downsampled Hi-C matrices. Results for chromosome 8 are shown. Hi-C contact matrices (**A**–**C**). Collinearity of Hi-C maps guided by the maize RefGen_v5 reference genomes (**D**–**F**). Collinearity of the Hi-C maps and their underlying guide maps. The header of each column reports the downsampling level, the number of Hi-C links after thinning, how many of these are on chromosome 8 (inside parenthesis), and the correlation coefficient between marker positions in the Hi-C map and the reference genome

Wet-lab procedures to generate Hi-C linkage information are possibly more demanding than those required for long-read sequencing. Short-read sequencing of Hi-C libraries constitutes a substantial fraction of the overall assembly cost (~ 30% for the homozygous 5 Gb genome of barley). That cost may be easily cut by sequencing Hi-C libraries less deeply but reducing coverage by too much may compromise map quality and entail delays, as additional sequencing data may have to be obtained. To understand how sparser Hi-C data impact map quality, we thinned the list of maize Hi-C links and made maps (minus the manual curation) from the downsampled data (Additional file 1: Fig. S5). Map quality did not worsen appreciably with ten times fewer links, i.e. 9 million of them. At extreme downsampling levels (1000-fold and more), Hi-C matrices start to show erroneous results and fail to detect assembly errors (Fig. 5C, F) and the correlation between Hi-C map and reference deteriorated (Additional file 1: Fig. S5). A useful rule of thumb for pangenome projects is to aim for 100 million raw reads per gigabase of haploid genome size in an inbred species, e.g. 200 million for a maize inbred. Downsampling as described here can tell if sequencing depth can be reduced in the next round of assembly. Allowance should also be made for heterozygous and polyploid organisms. Also bear in mind that the Hi-C read mapping process may remove up to 90% of the read pairs because they do not map uniquely (MAPQ < 10).

### Low-density guide maps are good enough

**Fig. 6:**
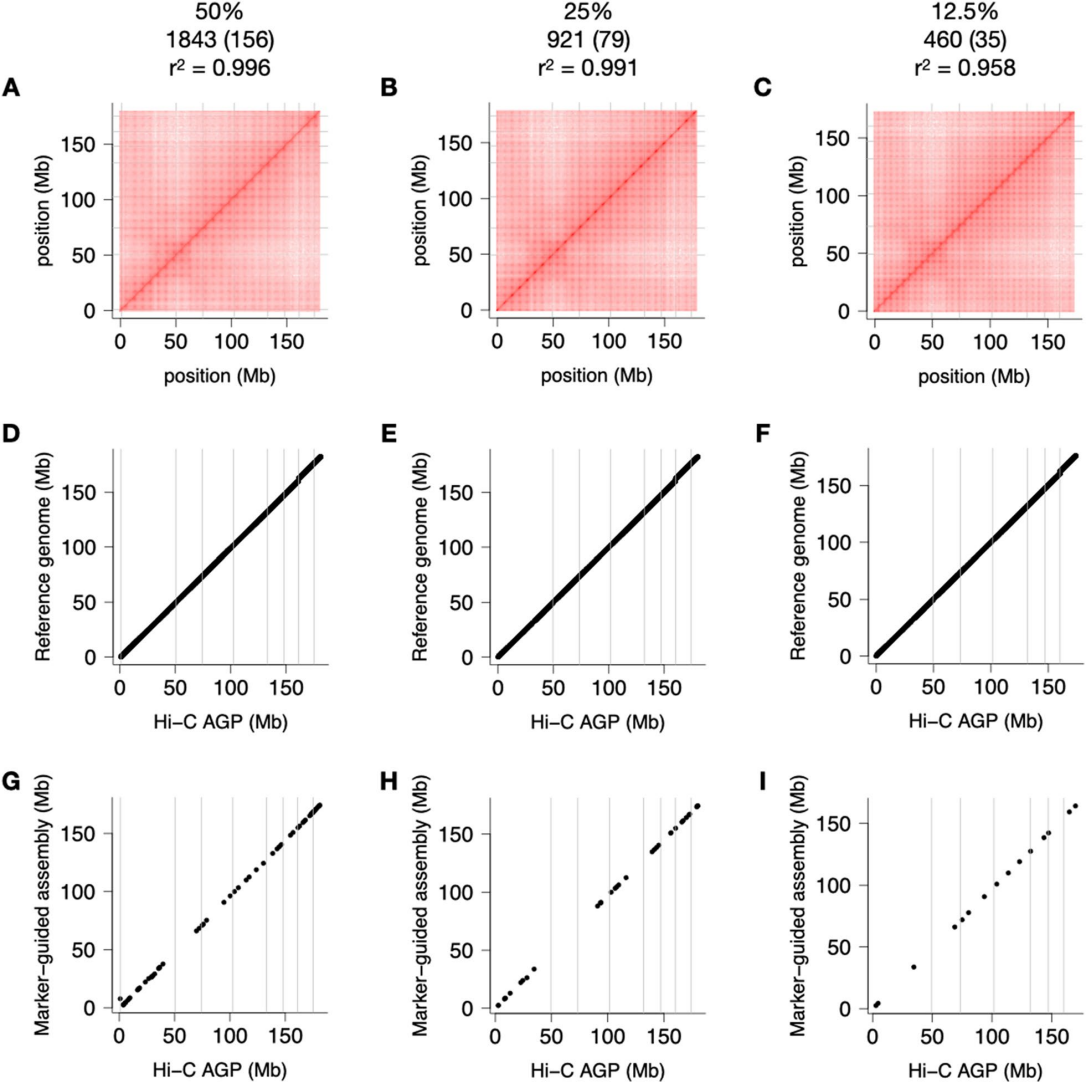
Impact of guide map density on assembly quality. Hi-C maps guided by genetic maps of different sizes were constructed. Results for chromosome 8 are shown. Hi-C contact matrices (**A**–**C**). Collinearity of Hi-C maps guided by the maize RefGen_v5 reference genomes (**D**–**F**). Collinearity of the Hi-C maps and their underlying guide maps (**G**–**I**). The headers of each column report the downsampling levels, the number of markers in the downsampled guide maps, the marker counts on chromosome 8 inside parenthesis, and the correlation coefficients between marker positions in the Hi-C map and the reference genome

We asked ourselves how dense a linkage map has to be to serve as a guide map for TRITEX. This is a pertinent question in species where no reference genome, and possibly few other genomic resources, are available and TRITEX is used to construct the very first genome sequence of a species. To assess how the density of the guide map affects the outcome of Hi-C scaffolding, we constructed uncurated Hi-C maps from subsets of different sizes of markers in the IBM guide map. We randomly selected 50%, 25%, and 12.5% markers from the IBM universe. On one hand, some chromosomes had correct Hi-C maps even if only 35 guide markers were placed on them (Fig. 6). On the other hand, some Hi-C maps left out or put contigs in the wrong places at higher downsampling levels (Fig. 6H and I; Additional file 1: Fig. S6). As a non-visual measure of Hi-C map quality, we computed the correlation coefficients between positions of contigs in the reference genome and downsampled Hi-C maps. Even when retaining only 1/8 of markers (a total of 460 and as little as 24 per chromosome), the correlation is still high (mean r^2^ = 0.958, minimum across 30 replications: 0.83). This level of discordance between an uncurated Hi-C map and the ground truth will require more manual effort on part of the user, but it will most likely not disrupt their ability to piece together accurate pseudomolecules. Importantly, the recommendations derived from downsampling Hi-C markers and guide-map markers are predicated on highly contiguous and mostly accurate primary assemblies as can be generated from contemporary long-reads platform. The picture may look different in fragmented short-read assemblies with more misassemblies.

## Discussion

TRITEX serves the practical needs of genome researchers to assemble and validate chromosome-scale genome sequences. Contig-level assemblies may be good enough for some applications such as guiding transcriptome assemblies or in-depth analysis of single genetic loci. But chromosome-scale sequences are indispensable for selection sweep scans and genotype-wide association mapping, which rely on linkage information to calculate or contextualize summary statistics. We anticipate that manual curation of genome assemblies will be needed for at least the next 3 to 5 years to construct and validate chromosomal pseudomolecules. Navrátilová et al. [9] have singled out one factor that can prevent telomere-to-telomere assembly in complex plant genomes: long homogenous satellite arrays with nucleotide compositions unfavorable to the PacBio platform. New technologies may overcome these obstacles. It is both conceivable and desirable that long read and complementary linkage data will in the future be gathered at multiplexing levels these days only seen in SNP chip or sequence-based genotyping. The hands-on work expended in TRITEX’s correct-map-inspect cycle would degrade into a tiresome chore and a major bottleneck. This predicament may be avoided by another conceivable and even more desirable development, that of ever longer and ever more accurate reads to underpin hands-off carefree telomere-to-telomere sequence assembly with negligeable error rates. Then TRITEX will go the way of short-read assemblers and retire into blissful obsolescence.

One main feature of TRITEX is the use of a genetic map to guide scaffolding, by assigning sequences to chromosomes. Other available software such as 3D-DNA [27], SALSA [38], and ALLHiC [39] rely only for Hi-C for clustering sequences into chromosomes. This can possibly lead to more rounds of manual curation and to difficulty in placing some sequences correctly on the chromosomes, especially on repetitive regions. The guide map mitigates these issues and still allow for the detection of structural variations. Nonetheless, the need for a genetic map is also a key impediment to the adoption of TRITEX in assembly projects across the tree of life. A preference for vegetative propagation, long generation times, difficult artificial crosses, few offspring resulting from such crosses, or eccentric meiosis in autotetraploid [40] may all make genetic mapping intractable. Gamete sequencing [41, 42] may alleviate some of these issues in certain species, but many taxa will remain recalcitrant. If no guide map can be had, meaning nothing at all is known about the number of chromosomes or the order of sequence tags on them, methods such as the mentioned above will be better suited.

The corresponding author of the present paper holds leading or advisory positions in cereal pangenome projects and is committed to using, maintaining, and updating TRITEX. Long-read TRITEX has been used to assemble the current reference genome sequence of barley (MorexV3, [2]) and sequence assembly wheat cv. Fielder [43]. The near future may see an extension of the TRITEX workflow to genomes in which chromosomes occur in multiple distinct, as opposed to identical, copies. *Hordeum bulbosum* is phylogenetically close related to inbreeding barley, the species that has motivated most of the TRITEX developments so far. It is outcrossing, highly heterozygous species with both diploid and autotetraploid cytotypes. The assembly for multiple *H. bulbosum* genomes may catalyze the implementation of TRITEX functions to create haplotype-resolved assemblies of higher-ploidy genomes.

## Conclusions

We have compiled a technical guide to the usage of TRITEX, a pipeline for assembling chromosome-scale plant genomes for de novo or pangenome projects. TRITEX uses long-read sequence assemblies and proximity ligation data plus a guide map to build chromosome pseudomolecules. The guide map is used to assign contigs to chromosomes, decreasing the amount of needed manual curation. Even so, manual curation is a crucial step, and one new feature of TRITEX is the simple and intuitive way of performing it, using plots and a user-editable table. Our pipeline has been used in several assembly projects and we anticipate plant scientists to use in future to construct reference and pangenome sequences of ever more species.

## Methods

### Data, HiFi assembly, and Hi-C mapping

We downloaded a PacBio HiFi dataset (SRA accession number SRR11606869) and a Hi-C dataset (SRA accession number PRJNA391551) for maize (*Zea mays*) variety B73. The maize reference maize references used in this study are available under accessions GCF_902167145.1 (RefGen_v5) and GCA_000005005.1 (AGP_v1).

HiFi assembly was performed with hifiasm v. 0.15.1-r334 [1] using default parameters. We converted the GFA output to FASTA with gfatools v. 0.4-r179 (https://github.com/lh3/gfatools). We in-silico digested (enzyme: *Mbo*I) the assembly using the TRITEX script “digest_emboss.zsh”, available at the TRITEX repository. Next, we mapped the Hi-C reads to the digested assembly using the script “run_hic_mapping.zsh”.

We followed two approaches to assemble the maize genome using TRITEX from the HiFi assembly generated in the previous step. The first one used a guide map with markers derived from the maize reference genome assembly available and the second one with markers from physical and genetic positions from a SNP array map of the Intermated B73xMo17 population [36].

### Reference-based assembly guide map

The reference-based guide map was created from the latest available maize B73 reference genome (RefGen_v5). Single-copy regions ≥ 100 bp were extracted from it using the TRITEX script “mask_assembly.zsh. Then, these single-copy regions were mapped to the HiFi assembly using minimap2 v. 2.17 with parameters “-I 20G” “-x asm5”, and “-2”.

### Marker-guided assembly guide map

The other strategy consisted of using the physical and genetic positions of markers from an Intermated B73xMo17 population. We extracted sequences 50 bp up- and downstream of the marker position in maize AGPv1 with the module “getfasta” from BEDTools v.2.30.0 [30]. Then, we mapped these sequences to the HiFi assembly using minimap2 v. 2.17 with same parameters as described above.

### Hi-C map construction

Hi-C map construction was done in R using TRITEX functions. The annotated code is available here: https://tritexassembly.bitbucket.io/.

### Downsampling analysis

To evaluate the impact of lower depth of Hi-C sequencing, we downsampled the Hi-C matrix by randomly removing variable fractions of Hi-C pairs from the list of Hi-C links in the assembly object. For each fraction (1/n, with n = 10, 25, 50, 75, 100, 250, 500, 750, 1000, 2500, 5000, 7500), we calculated the Pearson correlation between the contig positions in the reference-based vs. the downsampled assembly without the final step of manual curation. This procedure was repeated 30 times for each fraction.

Similarly, we reduced the number of guide map markers (IBM markers) to assess the impact of marker density on assembly quality. We retained randomly selected sets of markers, comprising 50%, 25%, and 12.5% of the total and ran the TRITEX reference-guide Hi-C scaffolding for each set. We calculated the Pearson correlation between the contig positions in the reference-based assembly and the assemblies guided by downsampled maps without any manual curation. This process was repeated 30 times for each downsampling fraction.

### Alignment of ribosomal DNA sequences

The sequence between 16,745,916 bp and 16,749,299 bp on chromosome 6 of maize B73 RefGen_v5 (one 45S rDNA monomer) was used as a query for BLAST alignment [44]. High-scoring pairs with e-values < 1e−10 were counted in 1 Mb genomic windows.

## Supplementary Information

**Additional file 1: Figure S1.** Example diagnostic plots of chimeric contigs that had to be broken. **Figure S2.** Screenshot of the Hi-C map inspector R Shiny app showing a chimera in a contig. **Figure S3.** Alignment of ribosomal sequence against the chromosome 6 pseudomolecule. **Figure S4.** Contact matrices and collinearity plots of all marker-based assembly pseudomolecules. **Figure S5.** Correlation between the downsampled assembly (number of Hi-C links) and the reference-based assembly. **Figure S6.** Correlation between the downsampled assembly (number of markers) and the reference-based assembly.

## Data Availability

The datasets analyzed during the current study are available in the NCBI SRA repository, under accession numbers SRR11606869, PRJNA391551, GCF_902167145.1, and GCA_000005005.1. Datasets generated during the current study are available in the e!DAL repository [45], (https://doi.org/10.5447/ipk/2022/20) [46]. The TRITEX code used in this manuscript is also available in e!DAL (https://doi.org/10.5447/ipk/2022/28) [47].
