# Supplementary material for: A technical guide to TRITEX, a computational pipeline for chromosome-scale sequence assembly of plant genomes
Source: Plant Methods. 2022 Dec 2;18:128. doi: 10.1186/s13007-022-00964-1 (PMC9719158; doi:10.1186/s13007-022-00964-1)
Supplement: Supplementary file 1 — Additional file 1: Figure S1. Example diagnostic plots of chimeric contigs that had to be broken. Figure S2. Screenshot of the Hi-C map inspector R Shiny app showing a chimera in a contig. Figure S3. Alignment of ribosomal sequence against the chromosome 6 pseudomolecule. Figure S4. Contact matrices and collinearity plots of all marker-based assembly pseudomolecules. Figure S5. Correlation between the downsampled assembly (number of Hi-C links) and the reference-based assembly. Figure S6. Correlation between the downsampled assembly (number of markers) and the reference-based assembly. [file 13007_2022_964_MOESM1_ESM.pdf]

Additional file 1

Supplementary figures

A technical guide to TRITEX, a computational pipeline for chromosome-scale sequence assembly of plant genomes

Marina Püpke Marone[1,2], Harmeet Chawla Singh[3,4], Curtis J Pozniak[3], Martin Mascher[1,5]

1: Leibniz-Institute of Plant Genetics and Crop Plant Research (IPK) Gatersleben, Seeland, Germany

2: University of Campinas, Department of Genetics, Evolution, Microbiology and Immunology, Campinas, Brazil

3: Crop Development Centre and Department of Plant Sciences, University of Saskatchewan, Saskatoon, SK, S7N 5A8, Canada

4: Department of Plant Science, University of Manitoba, Winnipeg, MB R3T 2N2, Canada

5: German Centre for Integrative Biodiversity Research (iDiv) Halle-Jena-Leipzig, Leipzig, Germany

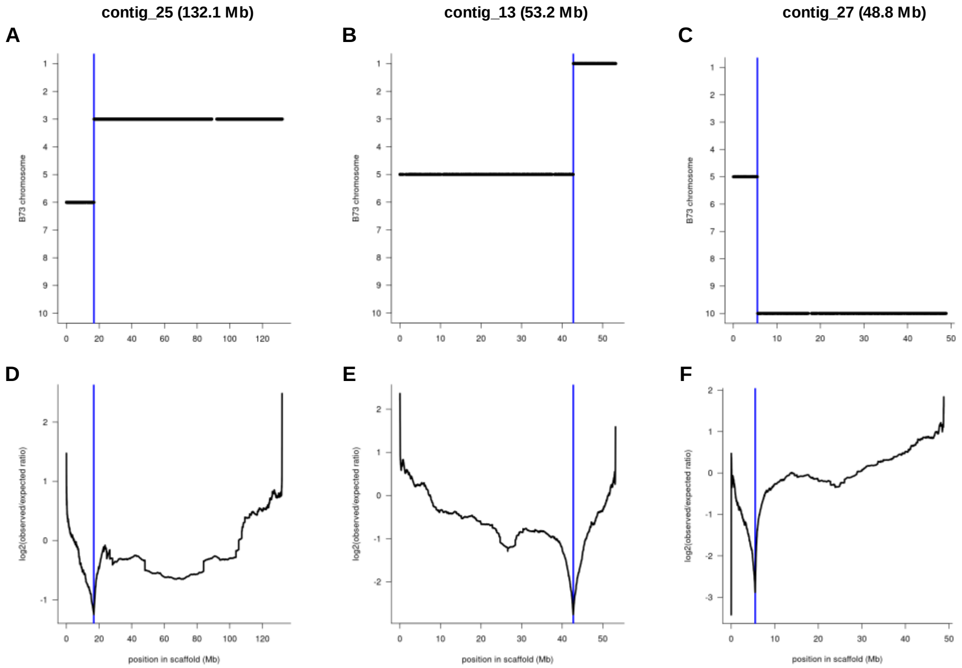

Figure S1: Example diagnostic plots of chimeric contigs that had to be broken. Each column shows one contig and the blue lines mark the breakpoints. **(A, B, C)** Positions of guide map markers assigned to chromosomes. **(D, E, F)** Physical coverage with Hi-C data (normalized by the respective contigs means) along the length of the contigs.

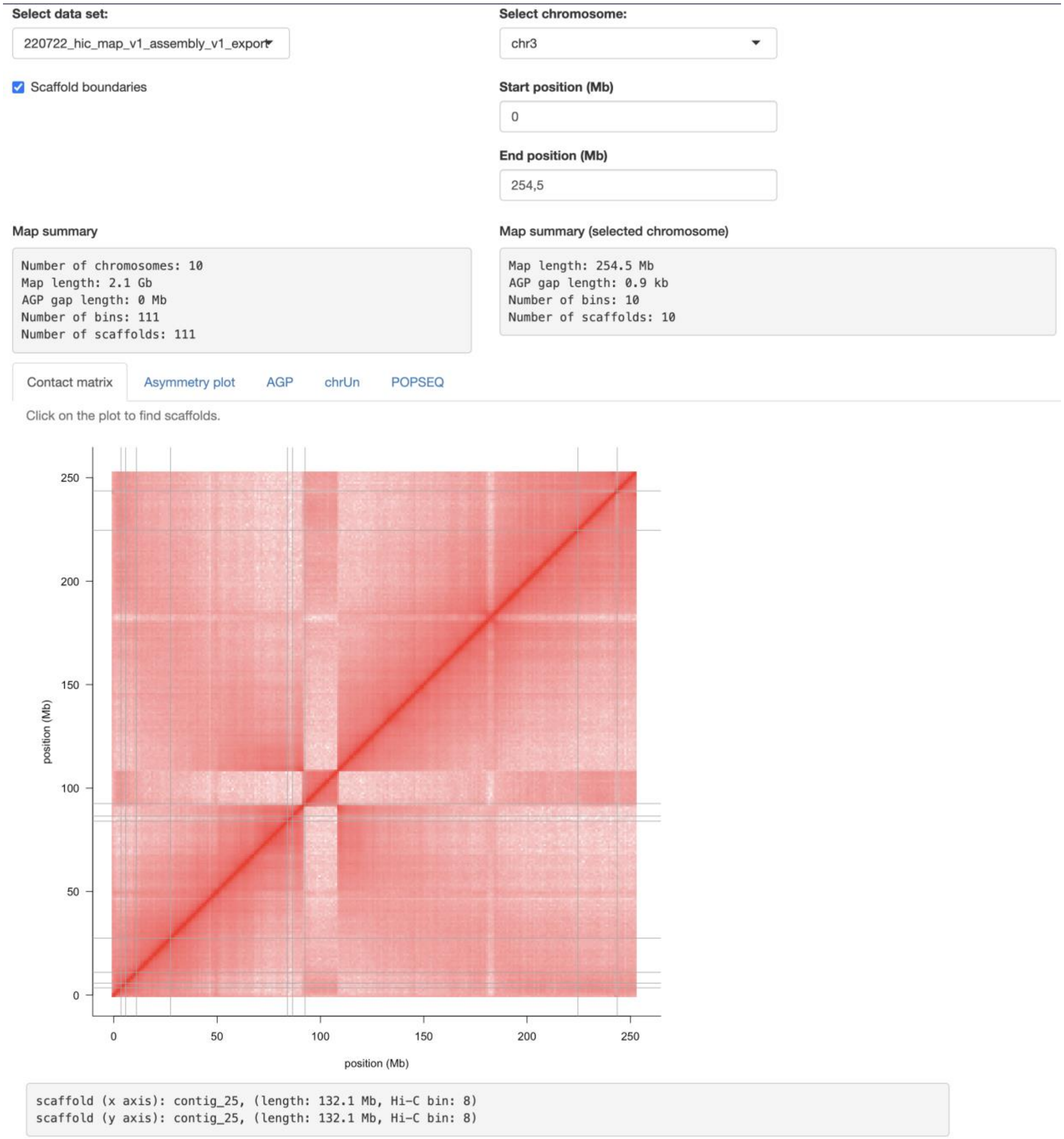

Figure S2: Screenshot of the Hi-C map inspector R Shiny app showing a chimera in a contig. Users can select and enlarge regions of Hi-C map and click on genomic regions containing chimeric scaffolds to get their names for further scrutiny and correction on the R command line. In this case, a chimera in contig_25 (see grey box below the contact matrix) is shown. Grey lines mark contig boundaries.

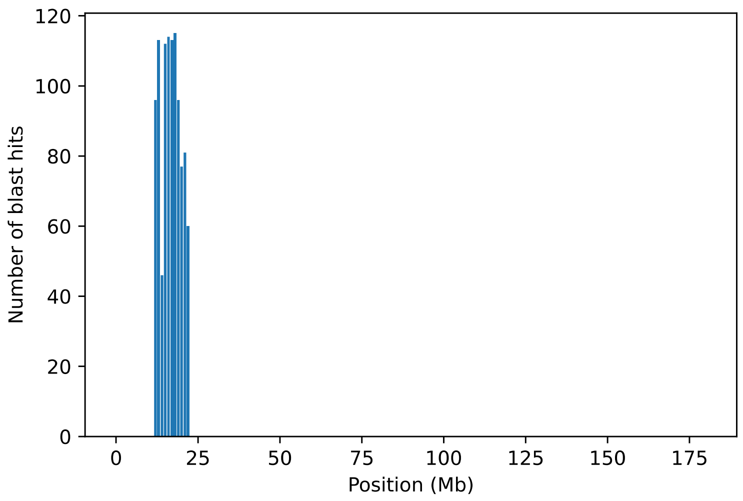

Figure S3: Alignment of ribosomal sequence against the chromosome 6 pseudomolecule. We performed a BLASTn using one repeat of the sequence of the maize B73 ribosomal locus (16,745,916 bp to 16,749,299 bp of chromosome 6) and counted the number of hits in 1 Mb windows.

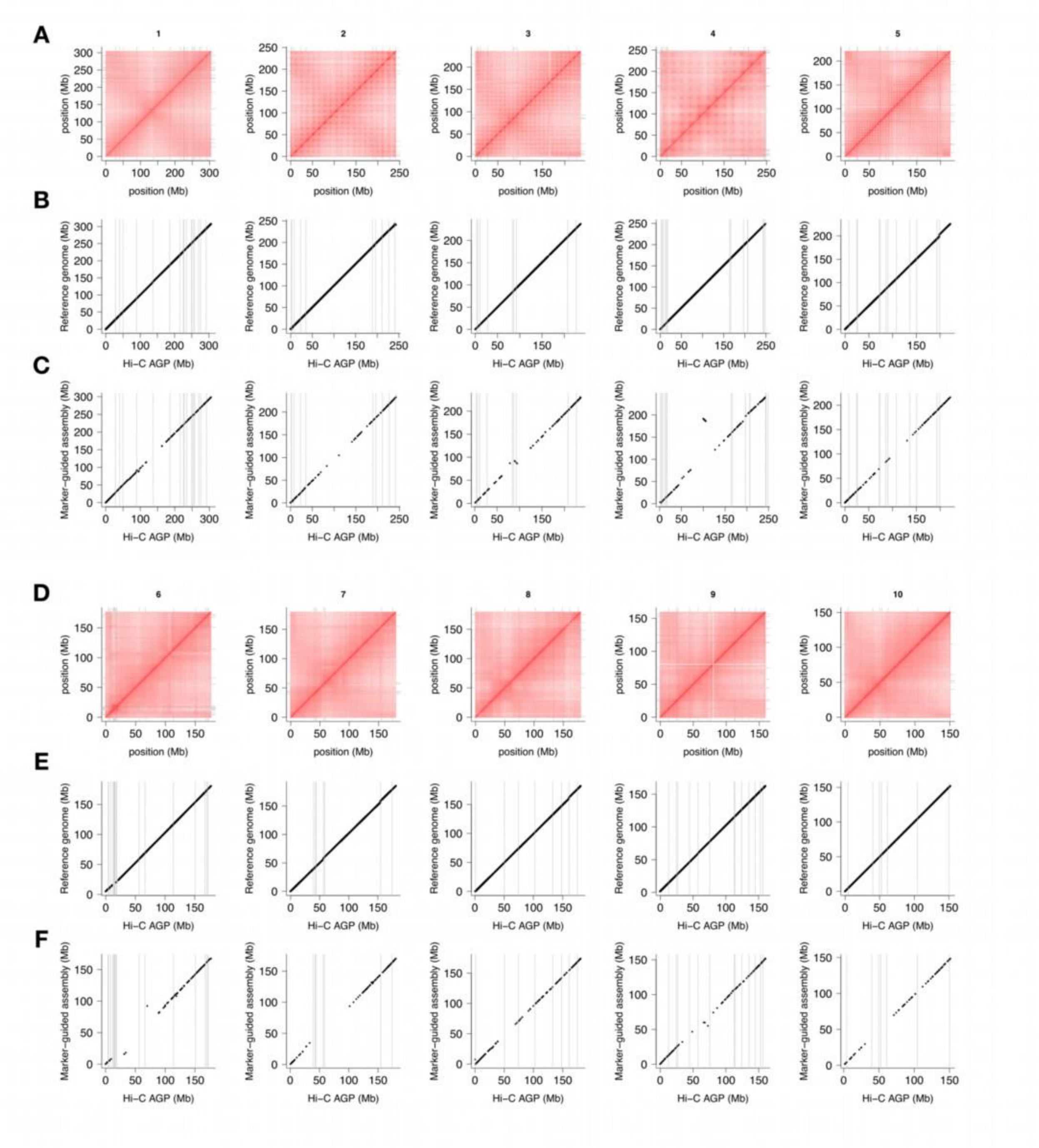

Figure S4: Contact matrices and collinearity plots of all marker-based assembly pseudomolecules. Hi-C contact matrices (**A and D**). Collinearity of Hi-C maps guided by the maize RefGen_v5 reference genomes (**B and E**). Collinearity of the Hi-C maps and their underlying guide maps (**C and F**).

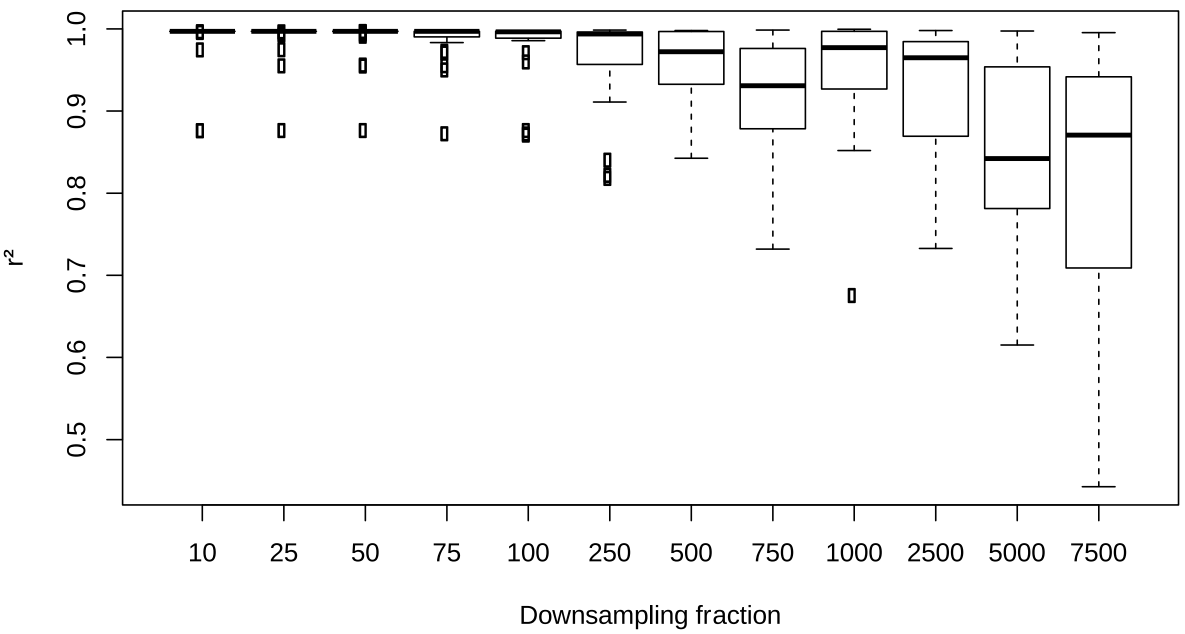

Figure S5: Correlation between the downsampled assembly (number of Hi-C links) and the reference-based assembly. We removed different fractions of Hi-C pairs and calculated the Pearson correlation between the contig positions in reference-based assembly and the downsampled ones. Data for 30 replications for each downsampling fraction are shown.

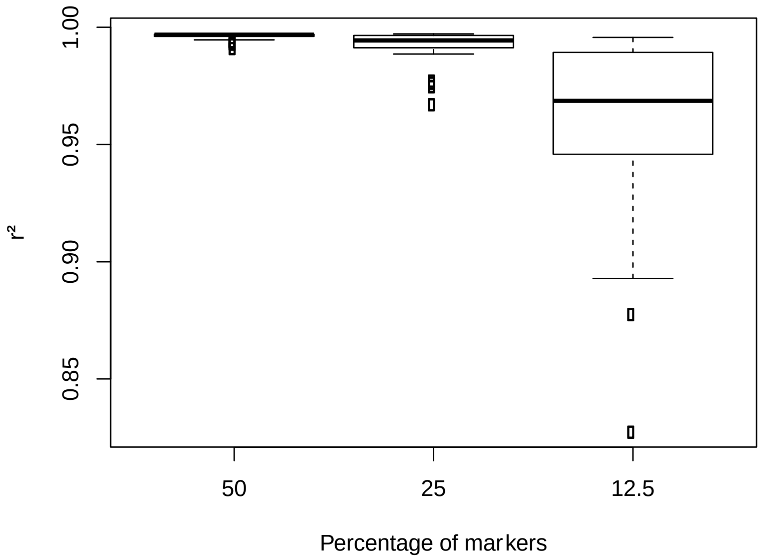

Figure S6: Correlation between the downsampled assembly (number of markers) and the reference-based assembly. We downsampled the number of markers in the marker-based assembly and calculated the Pearson correlation between the contig positions in reference-based assembly and the downsampled ones. Data for 30 replications for each downsampling fraction are shown.
